# A Role for the Amygdala in Impairments of Affective Behaviors Following Mild Traumatic Brain Injury

**DOI:** 10.3389/fnbeh.2021.601275

**Published:** 2021-03-04

**Authors:** Taylor A. McCorkle, Jessica R. Barson, Ramesh Raghupathi

**Affiliations:** ^1^Graduate Program in Neuroscience, Graduate School of Biomedical Sciences and Professional Studies, Drexel University College of Medicine, Philadelphia, PA, United States; ^2^Department of Neurobiology and Anatomy, Drexel University College of Medicine, Philadelphia, PA, United States

**Keywords:** mild TBI, depression, anxiety, posttraumatic stress disorder, basolateral amygdala, central amygdala, GABA, CRF

## Abstract

Mild traumatic brain injury (TBI) results in chronic affective disorders such as depression, anxiety, and fear that persist up to years following injury and significantly impair the quality of life for patients. Although a great deal of research has contributed to defining symptoms of mild TBI, there are no adequate drug therapies for brain-injured individuals. Preclinical studies have modeled these deficits in affective behaviors post-injury to understand the underlying mechanisms with a view to developing appropriate treatment strategies. These studies have also unveiled sex differences that contribute to the varying phenotypes associated with each behavior. Although clinical and preclinical studies have viewed these behavioral deficits as separate entities with unique neurobiological mechanisms, mechanistic similarities suggest that a novel approach is needed to advance research on drug therapy. This review will discuss the circuitry involved in the expression of deficits in affective behaviors following mild TBI in humans and animals and provide evidence that the manifestation of impairment in these behaviors stems from an amygdala-dependent emotional processing deficit. It will highlight mechanistic similarities between these different types of affective behaviors that can potentially advance mild TBI drug therapy by investigating treatments for the deficits in affective behaviors as one entity, requiring the same treatment.

## Introduction

Traumatic brain injury (TBI) is a serious public health concern that affects over 1.5 million people each year and results in the death and disability of thousands, with mild TBI comprising the greatest proportion of these cases (Kay et al., 1993; Coronado et al., 2011; McMahon et al., 2014). In the civilian population, mild TBI occurs largely because of playing contact sports such as American football, soccer, and ice hockey, whereas military personnel are exposed to mild blast TBI (Prins et al., 2013; Baldwin et al., 2018). Although sports-related concussions and mild blast TBI occur through different means, they share a similar prevalence of about 15–20% in civilian and veteran populations, as well as similar behavioral deficits (McKee and Robinson, 2014; Gardner and Yaffe, 2016).

Depression, anxiety, and fear/post-traumatic stress disorder (PTSD) are affective behavioral impairments that are among the most frequently reported behavioral problems that manifest in the chronic period following mild TBI (Baldassarre et al., 2015; Ellis et al., 2015; Horn et al., 2016; Bunt et al., 2020). The severity of these deficits is known to vary by sex, in that women are more prone to exhibiting affective disorders post-injury (Broshek et al., 2005; Bunt et al., 2020). Interestingly, the heightened response women exhibit following mild TBI is not consistently seen following moderate or severe TBI (Lavoie et al., 2017). Clinical studies used qualitative methods to diagnose the emotional state of an individual in conjunction with brain imaging tasks allowing for the identification of potential brain regions involved in affective disorders (Lange et al., 2016; Chong and Schwedt, 2018; Bunt et al., 2020; Teymoori et al., 2020). In preclinical models, validated behavioral assays have become the mode of quantifying what are usually thought to be subjective, affective states (Can et al., 2011, 2012; Calhoon and Tye, 2015; Eagle et al., 2016; Liu et al., 2018). Through these assays and the accompanied use of other biological techniques, mechanistic underpinnings of each disorder have been identified, as well as sex differences in deficit expression. However, limitations in the effectiveness of current drug regimens for affective disorders suggest that deficits following mild TBI should be examined in a new light (Xiong et al., 2013). Neurobiological studies have suggested that the amygdala is a key brain region involved in emotional processing (Phelps and Ledoux, 2005; Pessoa, 2010; Kim et al., 2011; Korgaonkar et al., 2019), and its impairment is associated with the manifestation of the affective disorders seen after TBI (Han et al., 2015; Hoffman et al., 2019). Understanding the mechanistic underpinnings of dysfunction in this brain region following injury will advance the field of TBI research. This review will discuss the current methods, corresponding mechanisms, and drug therapies used to identify and treat affective behavioral deficits in human and animal models of mild TBI. Because of the high comorbidity and mechanistic similarities across depression, anxiety, and fear, we suggest that they should be considered as a collective deficit in emotional processing stemming from impairment within the amygdala.

## Human Studies

### Definition and Epidemiology of Mild TBI

Mild TBI is often used interchangeably with concussion (Sussman et al., 2018) and affects approximately 15–20% of the population (McKee and Robinson, 2014; Gardner and Yaffe, 2016). Further, about 50% of people fail to report their injury suggesting that a majority of mild TBI incidents may be repetitive (Vargas et al., 2015; Pryor et al., 2016; Baldwin et al., 2018). Typically, mild TBI is defined by a Glasgow Coma Scale score between 13 and 15 along with a lack of, or very minimal, impairment in the level of consciousness. The two main types of mild TBI are contact-based such as sports-related concussions where some form of physical contact occurs, and blast-based which usually occurs as a result of exposure to an explosion (blast wave) during combat (Prins et al., 2013; Baldwin et al., 2018). Moreover, although it can happen at any age, mild TBI is particularly prevalent amongst adolescents who participate in contact sports (Baldwin et al., 2018), and young adults, who represent most enlisted military personnel (Lange et al., 2016). The risk for mild TBI is greater in boys and men due to sports including football and ice hockey and the fact that the military is a male-dominant field. However, girls are more prone to concussion when looking at gender-comparable sports (Baldwin et al., 2018).

### Behavioral Consequences of Mild TBI

Immediately after a concussion, patients experience headache, dizziness, nausea, sensitivity to noise, motor impairments, and deficits in executive functioning (Szczupak et al., 2016). Typically, these somatic signs and symptoms are documented using either the Sport Concussion Assessment Tool (SCAT) or the Immediate Post-concussion Assessment of Cognitive Testing (ImPACT). These acute symptoms resolve within 7-10 days in a vast majority of patients but can exist for 90 days or more in those that are then diagnosed with post-concussion syndrome (Ryan and Warden, 2003). In addition, and to a greater extent in those that have suffered multiple concussions, brain-injured patients can develop deficits in learning and memory and affective behavior disorders such as depression, anxiety and fear which can persist for years post-injury (Manley et al., 2017). Military personnel who suffer from mild blast TBI experience similar acute symptoms as individuals suffering from sports-related concussion and develop chronic problems such as impaired cognition (Waid-Ebbs et al., 2014), depression and post-traumatic stress disorder, which largely consists of anxiety and fear-related behaviors (Shively and Perl, 2012). In the chronic post-concussion phase, both cognitive and affective behaviors are assessed using more specific and sensitive measures such as the Wechsler Intelligence Scale, the Tower of London test, the Patient Health Questionnaire (PHQ), the Generalized Anxiety Disorder-7 (GAD-7) scale and the Clinician Assessment of PTSD each with its defined threshold for determining the severity of the deficit (Tables 1, 2). Although previous research has found that both sexes exhibit some degree of affective impairment, the consensus of literature states that women experience significantly exacerbated symptoms (Broshek et al., 2005; Sufrinko et al., 2017). Thus, the prevalence of depression, anxiety, and fear is increased in women compared to men (Bunt et al., 2020). Moreover, studies suggest that the severity of behavioral deficits in women depends on their menstrual phase (Bazarian et al., 2010; Stein, 2013; Wunderle et al., 2014). For example, women injured when their progesterone concentrations were high had lower health scores 1-month following injury (Wunderle et al., 2014). Another study showed that menstrual phase had no effect on concussion symptom severity (Mihalik et al., 2009); however, this study was conducted using healthy collegiate athletes. Combined, these limited and conflicting studies highlight the need for further investigation into the impact menstrual cycle has on behavioral consequences of mild TBI in women. Moreover, men also show a degree of affective impairment as well as significant cognitive deficits post-injury (Roberts et al., 2019). Since affective behavioral disorders have cognitive components to them, this can serve as a way through which men exhibit greater signs of psychological impairment.

**TABLE 1:** Clinical studies that evaluated cognition (such as executive function, memory, and concentration) and emotional (such as anxiety, depression, and PTSD) behaviors following mild TBI.

Study description	Behaviors evaluated	
	
	Cognitive	Emotional
Veterans (*N* = 10) diagnosed with repeated mild blast TBI (Waid-Ebbs et al., 2014)	Executive functions measured using Tower of London, Behavior Rating Index of Executive Function-Adult Version and Delis Kaplan Executive Function System	Depression using the Beck Depression Inventory II (> 29 = severe depression)
		PTSD using the PTSD Checklist-Military Version (> 50 = PTSD)
Veterans 18 years or older deployed in OIF/OEF conflicts (Baldassarre et al., 2015)	Poor concentration and difficulty making decisions using the Neurobehavioral Symptom Inventory	Depression using the Beck Depression Inventory II (score ≥ 17)
		Anxiety using the Beck Anxiety Inventory (score ≥ 8)
		PTSD using the ClinicianAdministered PTSD Scale (lenient (F1/I2), moderate (F1/I2 plus total severity ≥ 45), and stringent (F1/I2 plus total severity ≥ 65)
Veterans of OIF/OEF conflicts (*N* = 2235, Schneiderman et al., 2008)	Memory and post-concussion symptoms	PTSD using the PTSD Checklist (> 50 = PTSD)
Active-duty Marines and Navy Corpsmen (*N* = 825, Glenn et al., 2017)	DID NOT TEST	PTSD using the clinician administered PTSD Scale (one criterion A event, one cluster B symptom, two cluster C symptoms, and two cluster D symptoms) and Fear-Potentiated Startle
Former NFL players (Roberts et al., 2019)	Cognition-related QOL using QOL in Neurological Disorders: Applied Cognition	Depression and Anxiety using PHQ-4 (score ≥ 3 for each behavior)
Active semiprofessional and professional football players (Pryor et al., 2016)	DID NOT TEST	Depression using the Center for Epidemiologic Studies Depression Scale (score range from 0 = little/no depression to 60 = major depression)
NFLPA retired players section (*N* = 1617, Schwenk et al., 2007)	DID NOT TEST	Depression using PHQ-9 (0–9 = no-to-mild; 10–27 = moderate-to-severe)
Male and female patients (*N* = 491) ages 12–18 with a diagnosed SRC within 30 days of a clinic visit (Bunt et al., 2020)	Memory and concentration using the SCAT-5 scale	Depression using GAD-7 Anxiety using PHQ-9
Male and female collegiate athletes (*N* = 84) with concussion (Vargas et al., 2015)	Reading, memory and concentration using the Wechsler Test of Adult Reading and the ImPACT score	Depression using the Beck Depression Inventory-Fast Screen (score ≥ 4)
Pediatric patients (19 and younger) referred to Pan Am Concussion Program (Ellis et al., 2015)	DID NOT TEST	Depression and Anxiety using the PCSS emotional sub scores (range of 0–24)
Mild TBI patients (18 years or older, *N* = 238) from the Northern California TBI Model Systems of Care database (Lavoie et al., 2017)	DID NOT TEST	Depression using PHQ-9 (0-9 no-to-mild, 10–27 moderate-to-severe)
Mild TBI patients (18 years and older) following their first head injury ages (Rao et al., 2010)	Attention, learning, delayed recall and memory using the MMSE, National Adult Reading Test and the Hopkins Verbal Learning Test-Revised	Depression using the Structured Clinical Interview for DSM-IV Axis 1 disorders
		Social Functioning Examination
Patients (18 years and older) who sustained TBI at least 3 months prior (*N* = 101, Mohammad Farris Iman Leong Bin Abdullah et al., 2018)	DID NOT TEST	Depression and Anxiety using the Structured Clinical Interview for DSM-IV Axis I Disorders Research Version
Patients (16 years or older) who sustained a TBI at least 6 months prior (Teymoori et al., 2020)	DID NOT TEST	Depression and anxiety using the PHQ subsection for Anxiety and Depression (scores of 8 and 10 used as cutoff, respectively)
		Anxiety using the GAD-7 (score of 8 used as cutoff)
		PTSD using the PTSD Checklist for DSM-V (score of 33 used as cutoff)

*Whereas certain studies evaluated both sets of behaviors, a few only reported the incidence of emotional behaviors. The studies described in this table did not assess structural alterations in the brains of mild TBI patients.*

*GAD-7, Generalized Anxiety Disorder-7; ImPACT, Immediate Post-concussion Assessment and Cognitive Testing; MMSE, MiniMental State Examination; OIF/OEF, Operation Iraqi Freedom/Operation Enduring Freedom; PCSS, Post-Concussion Symptom Scale; PHQ, Patient Health Questionnaire; PTSD, Post-Traumatic Stress Disorder.*

**TABLE 2:** Clinical studies that evaluated structural/functional alterations (using imaging techniques) in patients that were tested for cognitive (such as executive function, memory, and concentration) and emotional (such as anxiety, depression, and PTSD) behaviors following mild TBI.

Study description	Structural/functional changes		Behavioral changes	
		
	Amygdala	Other regions	Cognitive	Emotional
Combat veterans from Iraq or Afghanistan with mild TBI only (N = 15) or mild TBI + PTSD (*N* = 17, Shu et al., 2014)	ND	Event-related potentials in the dorsal anterior cingulate cortex	DID NOT TEST	Depression using the Beck Depression Inventory II
				PTSD using the clinician administered PTSD Scale (>65)
US Service Members (*N* = 153, Tate et al., 2016	Changes in shape (using MRI) of right anterior amygdala as a function of time after injury	Changes in surface areas (using MRI) of anterior medial accumbens and left anterior medial caudate Increased	Memory and decision-making using the Neurobehavioral Symptom Inventory	PTSD using the clinician administered PTSD Scale (DSM-IV Criteria)
Football players (N30) with acute concussion (Mustafi et al., 2018)	ND	Increased mean diffusivity (using DTI) in corpus callosum, superior longitudinal fasciculus and corona radiata	SCAT	Depression and Anxiety using Brief Symptom Inventory
Active and former professional rugby players with history of concussion (*N* = 24, Wojtowicz et al., 2018)	Smaller left amygdala volumes using MRI	Smaller bilateral hippocampi in the absence of differences in whole brain cortical thickness using MRI	Learning and memory using the Rey Auditory Verbal Learning Test, the Rey Complex Figure Test and the Long Delay Recall Task	Depression, Anxiety, and Stress (comprised of 7-item scales)
				Alcohol Use Disorders Identification Test
Patients with mild TBI (*N* = 42, Giguère et al., 2019)	ND	ND	Executive function and working memory using Repeatable Battery for the Assessment of Neuropsychological Status and the Delis-Kaplan Executive Function System ColorWord inference test and the Digit Span task	Hospital Anxiety and Depression Scale (pathological scores are 11–21)
Chronic TBI patients (*N* = 54, Han et al., 2015)	Enhanced bilateral amygdala connectivity (using MRI) in TBI patients exhibiting depression group	No change in somato-motor cortex connectivity using MRI	Memory and executive functions using Immediate and Delayed Recall Tasks, the Full Scale Intelligent Quotient-2, the Wechsler Abbreviated Scale of Intelligence, the Wechsler Test of Adult Reading, and the Delis-Kaplan Executive Function System	Depression using the Beck Depression Inventory II (score up to 13 = minimal depression and 14–63 = mild to severe)
				PTSD using PTSD Checklist Stressor specific for DSM-IV
Patients with closed head injury (*N* = 91, Jorge et al., 2004)	ND	Volumes for the entire brain, the orbitofrontal cortex, medial-frontal cortex and lateral prefrontal cortex determined using MRI	Learning, memory, executive functions using the MMSE, the Rey Auditory Verbal Learning Test, the Rey Complex Figure Test and the Multilingual Aphasia Examination	Depression, anxiety, aggression
				Two semistructured interviews (Present State Examination) along with Structured Clinical Interview for DSM-IV diagnoses
				Hamilton Depression Rating Scale
				Hamilton Anxiety Scale
				Overt Aggression Scale

*Most studies that have evaluated structural/functional changes using advanced imaging techniques have focused on the cortex and hippocampus along with various white matter tracts. A few studies have evaluated alterations in structure and function of the amydgala.*

*DTI, Diffusion Tensor Imaging; MRI, Magnetic Resonance Imaging; PTSD, Post-Traumatic Stress Disorder; SCAT, Sport Concussion Assessment Tool.*

A search of clinical studies in PubMed using search terms such as mild TBI and cognition or mild TBI and depression or anxiety or fear revealed almost twice as many publications focused on cognitive impairments after mild TBI. However, recent studies revealed that patients develop depression, anxiety, and fear years after injury, which greatly contributed to a decreased quality of life (Baldassarre et al., 2015; Giguère et al., 2019; Tables 1, 2). Affective disorders are reported in approximately 20% of brain-injured individuals (Schwenk et al., 2007; McKee and Robinson, 2014; Scholten et al., 2016; Brassil and Salvatore, 2018), and can impact other behavioral outputs. For example, depression increases the bias to aversive memory recall (Leal et al., 2017), anxiety decreases the ability to handle stress as it directly involves the hypothalamic-pituitary-adrenal (HPA) axis (Maeng and Milad, 2015), and the expression of fear is linked to aggression (Gao et al., 2014).

Post-traumatic depression is characterized by alterations in affect, diminished interest in pleasurable activities, negative and intrusive thoughts, reductions in physical movement, decreased executive functioning, and loss of energy (Lavoie et al., 2017). Depression is evaluated based on the results from the Beck Depression Inventory II, the PHQ-9, or the Structured Clinical Interview for DSM-IV/V Axis 1 Disorders (criteria are described in Tables 1, 2). Approximately 15% of individuals with mild TBI and mild blast TBI suffer from post-injury depression (Schwenk et al., 2007; McKee and Robinson, 2014). This is a gross underestimation because more than 50% of individuals with a mild TBI fail to report symptoms (Rao et al., 2010; Vargas et al., 2015; Pryor et al., 2016). About 21% of individuals with mild TBI experience some form of anxiety disorder within the first year of injury which increases to 36% after the first year due to high long-term prevalence (Scholten et al., 2016); anxiety was measured using the Beck Anxiety Inventory, the GAD-7 scale or the PHQ (criteria are described in Tables 1, 2). The manifestation of fear-related behaviors (“avoiding similar situations”) is a hallmark of PTSD which also includes difficulty sleeping and flashbacks (Brewin et al., 2017). In the civilian population, PTSD occurs at a prevalence rate of less than 25%, whereas over 35% of military personnel exposed to mild blast TBI exhibit symptoms of PTSD (Schneiderman et al., 2008; Shu et al., 2014; Brassil and Salvatore, 2018). Individuals exposed to mild blast TBI experience heightened stress responses and emotional memory associations due to the nature of their situation. In addition, repeated traumatic events occurring during deployment results in strong, conditioned fear learning and expression of fear which may be the basis for the high risk of PTSD in this group (Glenn et al., 2017). Tests used to detect PTSD and their criteria are described in Tables 1 and 2.

Depression, anxiety, and fear-related behaviors following mild TBI are comorbid conditions (Tables 1, 2; Jorge et al., 2004; Ellis et al., 2015; Mohammad Farris Iman Leong Bin Abdullah et al., 2018; Teymoori et al., 2020). A study of over 100 individuals with a mild TBI used a Structured Clinical Interview for DSM-IV axis I disorders to determine that 25% showed signs of depression and 14% had anxiety, with fear-evoked PTSD representing a major contributor to this anxiety. Importantly, about half of these individuals had comorbid anxiety and depression (Mohammad Farris Iman Leong Bin Abdullah et al., 2018). In another study, the same structured interview was utilized, as well as the Hamilton Depression and Anxiety Scales to show that 33% of mild TBI patients exhibited symptoms of depression within the first year and within this group, 76% also had anxiety (Jorge et al., 2004).

### Structural Changes Following Mild TBI

The primary structural pathology of mild TBI is damage to white matter tracts. Post-concussion white matter changes are largely present in the frontal, parietal, and temporal lobes of the brain (Chong and Schwedt, 2018). An advanced MRI study demonstrated significantly altered diffusion properties of white matter tracts in concussed athletes, which were correlated with acute symptoms post-injury and functional deficits. Specifically, an increase in mean diffusivity in the corpus callosum, superior longitudinal fasciculus, and the corona radiata of the concussed group compared to healthy controls has been reported (Mustafi et al., 2018). Structural impairment can persist in adolescents and individuals who suffer multiple concussions, contributing to prolonged behavioral impairment (Chong and Schwedt, 2018). Furthermore, a study investigating former high school athletes that suffered repeated concussions showed higher mean diffusivity in the anterior limb of the internal capsule over 20 years post-injury (Terry et al., 2019).

Functional MRI studies have shown changes in hippocampal connectivity following repeated collisions in collegiate football players (Slobounov et al., 2017; Wojtowicz et al., 2018). A study of professional rugby players with a history of concussion demonstrated no significant differences between cortical thickness, but smaller whole brain hippocampus and left amygdala volumes compared to healthy controls (Wojtowicz et al., 2018). Neuroimaging studies of mild blast TBI patients tend to be understudied; however, one study showed that injury to the hippocampus in veteran populations results in globally decreased gray matter volumes compared to controls (Bhattrai et al., 2019). Alterations in hippocampal connectivity relate directly to cognitive and affective behavior deficits (Belujon and Grace, 2011; Leal et al., 2017). In military personnel who had suffered at least one mild TBI, the radial distance of the amygdala was positively correlated with time since injury, meaning individuals further removed from injury showed greater amygdala thickness (Tate et al., 2016). Moreover, imaging performed on individuals with moderate TBI and depressive symptoms demonstrated that these individuals have increased amygdala connectivity relative to healthy controls, and this increased connectivity was associated with affective disorders (Han et al., 2015). The amygdala is a key hub of emotional processing (Phelps and Ledoux, 2005; Pessoa, 2010; Kim et al., 2011; Korgaonkar et al., 2019), therefore, changes in its size or connectivity can lead to affective behavior impairments seen post-injury (Han et al., 2015; Hoffman et al., 2019). Structural impairments in the hippocampus and amygdala post-injury provide avenues through which affective behavioral disorders can manifest in individuals; however, these structures are not widely investigated in neuroimaging studies following mild TBI. Sex differences in the structural impairments following mild TBI are also understudied and filling this gap in literature would aid in understanding the relationship between behavioral outcome and structural alterations. Most studies that have evaluated chronic behavioral deficits in concussed patients typically do not provide assessment of structural or functional alterations (Table 1). The preponderance of studies that have included imaging correlates for behavioral changes focus on structures that may be more vulnerable to concussions such as white matter tracts and cortical areas at or near the sites of impact (Table 2), making it difficult to determine specific relationships between behaviors and intracerebral alterations.

## Animal Studies

### Definition and Models of Mild TBI

Multiple animal models of mild TBI that effectively reflect structural changes (axonal injury and neurodegeneration) and impairments in motor, cognitive, and affective behaviors typically reported in brain-injured clinical populations, have been developed (Malkesman et al., 2013; Bodnar et al., 2019). Mild TBI can be induced through impact to the intact skull, head, or exposure to a blast wave. Impact to the intact skull by way of an extended piston tip or a weight drop that does not result in skull fracture or hematoma is defined as “mild” (Ma et al., 2019). Because both sports-related concussions and mild blast TBI are characterized by their repetitive nature, the greatest challenge in developing a clinically appropriate animal model of contact or blast TBI is selecting the best approach and variability across the animal models exists in impact site, frequency, and severity (Weber, 2007). When performing repetitive mild TBI, impact frequency must be translational, realistically modeling the frequency in the human condition. Moreover, despite the immense variability that can exist between models of mild TBI, validation must be based on the structural and behavioral changes of mild TBI seen in human populations. To reduce variability in the site and magnitude of impact, animals are typically restrained and therefore require anesthesia such as isoflurane or ketamine (Rowe et al., 2014). Mild blast TBI has been performed with (Rowe et al., 2014) or without (Uddin et al., 2019) anesthesia, using a shock tube and cranium only blast injury apparatus, respectively (Kuehn et al., 2011; Ma et al., 2019; Uddin et al., 2019). Anesthetized animals exhibit similar behavioral and structural deficits as seen in awake rodent and clinical mild TBI models; therefore, the protective effects of the anesthetics are often minimal, but must be considered when deciding on which preclinical mild TBI model to utilize (Rowe et al., 2014). Moreover, it is important to note that the use of anesthesia limits complete fidelity of animal models to reflect concussion in humans, as humans are not anesthetized during injury.

### Behavioral Changes Following Mild TBI

Preclinical models of mild TBI exhibit short-and-long-term behavioral deficits post-injury. Acutely, brain-injured animals show impaired locomotion, working memory, and anxiety (Wright et al., 2017). Repetitive brain injury has been shown to lead to chronic deficits in social behavior, spatial working memory, spatial learning, and other prefrontal cortex and hippocampal-associated functions (Cheng et al., 2014; Nolan et al., 2018). The worsening or development of affective behavioral deficits is frequently reported in preclinical models of single and repetitive TBI (Teutsch et al., 2018; Popovitz et al., 2019; Beitchman et al., 2020), and mild TBI, and include spatial memory impairments, depression, anxiety, and impairments in fear-related behaviors, as seen in clinical populations (Petraglia et al., 2014; Wright et al., 2017). Moreover, animals subjected to repetitive brain injuries are at an increased risk of experiencing prolonged behavioral impairments post-injury (Petraglia et al., 2014). Affective behavioral impairments are also understudied in preclinical models of mild TBI, which often highlight the presence of these disorders without uncovering their mechanistic basis or examining potential therapies. Due to these issues, there are numerous gaps in the field of post-traumatic depression, anxiety, and fear, and these gaps will be explored in upcoming sections of the review.

Male and female brain-injured animals demonstrate impairment in both cognitive and affective behaviors, but male animals typically exhibit exacerbated cognitive impairments and female brain-injured animals show exacerbated affective disorders (Wright et al., 2017). TBI disrupts the female estrous cycle, causing imbalances in estrogen and progesterone levels and makes female animals more vulnerable to the negative effects of stressors (Wright et al., 2017; Fortress et al., 2019). Preclinical data investigating the effects of the estrous cycle on injury-induced behavioral outcomes are more extensive and conclusive than the current clinical data. Despite this knowledge on sex differences in behavioral outcomes, most preclinical models of mild TBI solely utilize male animals. However, although sex differences in structural and behavioral outcome of mild TBI are historically understudied, they have been gaining more attention in recent years.

### Pathology

Structural changes observed in animals following mild TBI closely reflect those seen in humans exhibiting minimal damage, mostly present as traumatic axonal injury in white matter tracts (Mierzwa et al., 2015; Kikinis et al., 2017; Hoogenboom et al., 2019). Imaging studies demonstrate decreased mean diffusivity in the genu of the corpus callosum and increased fractional anisotropy in white matter tracts in injured animals one-week post-injury (Kikinis et al., 2017; Hoogenboom et al., 2019) as well as decreased axial diffusivity in the corpus callosum 2 weeks-post repetitive mild TBI (Wright et al., 2017). Diffusion tensor imaging showed decreased expression of myelin basic protein in the corpus callosum and high fractional anisotropy in white matter tracts immediately after injury which are normalized in the months following injury in most cases (Herrera et al., 2017). MRI in a mouse model of repetitive mild TBI demonstrated microgliosis in white matter tracts acutely post-injury (Robinson et al., 2017). Sex differences in pathology following mild TBI are largely understudied. However, one study demonstrated that following single and repetitive mild TBI, male animals showed increased astrocyte reactivity in the corpus callosum only after mild TBI, whereas female animals showed increased reactivity only following repetitive mild TBI (Wright et al., 2017). The results of this study would suggest that potential sex differences in pathology should be further investigated.

Preclinical studies have also investigated structural deficits in regions such as the hippocampus and amygdala (Table 3). Single and repetitive closed head injury in young adult rats found significant changes in fractional anisotropy in the central amygdala (CeA, Kulkarni et al., 2019). These changes were seen at 7-8 weeks post-injury and were associated with impairments in affective behavior regulation. This study also demonstrated that repetitive mild TBI showed greater amygdala alterations than single injury; however, both injury models exhibited impairments compared to controls (Kulkarni et al., 2019). Moderate TBI in adult male rats resulted in dendritic hypertrophy in the basolateral (BLA) amygdala at 7- and 28-days post-injury, demonstrating the long-term structural alterations of the amygdala (Hoffman et al., 2017). Increased dendritic spine density in the amygdala, as well as increased dendritic branching in dendrites further from the soma regions have been found in a mouse model of mild blast TBI. This may be indicative of increased communication within amygdala circuitry following blast injury (Ratliff et al., 2019). Repeated mild blast TBI has shown alterations in the transcriptome within the BLA and CeA subregions which were associated with anxiety-like behaviors (Blaze et al., 2020), and single blast injury has led to a decrease in the number of BLA pyramidal neurons (Heldt et al., 2014). An impairment in inhibitory transmission in the BLA following mild TBI as indicated by a decrease in GABAergic neurons has also been reported (Almeida-Suhett et al., 2014) as well as decreased network excitability in the amygdala (Palmer et al., 2016). Repeated mild TBI was associated with decreased microglia in the hippocampus and BLA of brain-injured animals (Cheng et al., 2019) and hypoconnectivity in the hippocampus (Kulkarni et al., 2019). These studies highlight the need to further investigate both circuit-based dysfunction (e.g., alterations in the excitatory/inhibitory balance) and cellular alterations (neuronal damage, glial activation) within the various regions of the amygdala.

**TABLE 3:** Structural (using histology and/or imaging) and behavioral (cognition, anxiety, depression, fear) alterations in animal models of mild TBI.

Study description	Structural alterations		Behavioral alterations	
		
	Amygdala	Other regions	Cognitive	Emotional
Single or repeated mild TBI in adult male rats (Kulkarni et al., 2019)	Central amygdala altered diffusivity following single and repeated impact using diffusion weighted imaging	Changes in diffusivity in the caudate putamen, white matter, basal ganglia, brainstem, cerebellum. Hypoconnectivity in the hippocampus, midbrain dopamine system, hyperconnectivity in olfactory system	Novel Object Recognition and Barnes maze test to demonstrate deficits in working memory and spatial memory	DID NOT TEST
Midline fluid percussion brain injury in adult male rats (Hoffman et al., 2017)	Dendritic hypertrophy and activated astrocytes in the basolateral amygdala	Neurodegeneration and axonal injury in the cortex, hippocampus, thalamus and corpus callosum	DID NOT TEST	DID NOT TEST
Mild blast TBI in adult male mice (Ratliff et al., 2019)	No difference in dendritic length but increased spine density and branching at distal dendrites of amygdala neurons	ND	Deficits in spatial working memory using Novel Object Recognition Test	Anxiety-like behavior using the Staircase Test
Repeated mild blast TBI in adult male rats (Blaze et al., 2020)	Alterations in transcriptome within the BLA and CeA subregions in brain-injured rats	ND	No deficits in working memory using Novel Object Recognition	Anxiety-like and fear behaviors using the Light/Dark Box, Elevated Zero Maze, and Fear Conditioning tests
Repeated mild TBI in adult male wild-type and APP/PS1 mutant mice (Cheng et al., 2019)	Reduction in microglia within the BLA of brain-injured mutant mice	No difference in microglial reactivity between WT and mutant mice in the PFC, parietal cortex, corpus callosum, optic tract-white matter. Reduced microglial reactivity in hippocampus of mutant mice	Impaired spatial learning using the Barnes Maze	Risk-taking behaviors using the Elevated Plus Maze Impaired fear memory using the Passive Avoidance Test
Mild TBI in adolescent male rats (Almeida-Suhett et al., 2014)	Loss of GAD67-positive neurons in BLA of injured animals and impairment in inhibitory transmission in the BLA	ND	DID NOT TEST	Anxiety-like behaviors using the Open Field Test
Mild blast TBI in adult male C57BL/6 mice (Heldt et al., 2014)	No evidence of reactive glia in the BLA of brain-injured Neuronal loss in the BLA	No neuronal loss in cerebral cortex or striatum	DID NOT TEST	Anxiety using Open Field test Depressive behaviors using Tail Suspension Fear related behaviors using Acoustic Startle, Test, Prepulse Inhibition, Fear acquisition and extinction tests
Lateral fluid percussion brain injury in adult male C57BL/J6 mice (Palmer et al., 2016)	Decreased evoked EPSPs in the BLA Decreased network excitability in lateral amygdala	ND	DID NOT TEST	Impairments in fear acquisition using Cued Fear Conditioning

## Current Treatment Strategies for Post-Traumatic Affective Disorders

### Human Studies

Current treatment strategies for depression, anxiety and fear/PTSD in brain-injured patients utilize best practices that are in place for treating these disorders in patients that have not suffered a TBI (Plantier and Luauté, 2016; Gupta et al., 2019; Silverberg and Panenka, 2019). Thus, serotonin reuptake inhibitors (SSRIs) are the most utilized therapy for post-traumatic depression (Kraus et al., 2017; Silverberg and Panenka, 2019). However, a meta-analysis of clinical trials using SSRIs following concussion found that these drugs have no benefit for post-traumatic depression in some cases, but rather their effects stem from robust placebo effects (Silverberg and Panenka, 2019). Alternate therapeutic strategies include cognitive behavioral therapy (CBT) which helps modify negative thought patterns and has been used extensively in military veterans (Cooper et al., 2015; Fann et al., 2015; Ponsford et al., 2015). In addition to CBT, which is focused on information processing and memory, motivational interviewing has also been used on brain-injured military veterans suffering from PTSD and civilian populations with post-traumatic anxiety (Cooper et al., 2015). Melatonin, which can improve sleep quality following mild TBI, has been effective in attenuating anxiety in brain-injured individuals (Grima et al., 2018). Sedatives such as benzodiazepines are used to treat anxiety in individuals with mild TBI by working as positive allosteric modulators that increase the activity of the GABA-A receptor although the effects of benzodiazepines are transient and variable (Flower and Hellings, 2012; Plantier and Luauté, 2016).

### Animal Studies

Therapies for affective disorders that are currently utilized on brain-injured patients have typically not been tested in clinically relevant animal models of TBI (Cryan et al., 2002). Rather, pharmacologic interventions aimed at reducing acute neurodegeneration after TBI use depression, anxiety, and fear behavior as outcome measures. For example, both hyperbaric oxygen therapy and lithium (which inhibits the activity of the apoptosis regulator glycogen synthase kinase-3) have been used in animal models of TBI to reduce depression-like symptoms (Shapira et al., 2007; Lim et al., 2017). Similarly, antagonists of either the glutamate receptor, glucocorticoid receptor, or the corticotropin releasing factor (CRF) receptor 1 ameliorated post-traumatic anxiety-like behaviors (Fox et al., 2016; Kosari-Nasab et al., 2019) or PTSD-like behaviors (Perez-Garcia et al., 2018). Together with the clinical data, the pre-clinical studies highlight the disconnect between the bench and the bedside when it comes to treating affective disorders in TBI patients.

## An Integrated Approach to Post-Traumatic Affective Behaviors: The Role of the Amygdala

### The Significance of the Amygdala

Human and animal studies of mild TBI have demonstrated that affective behavioral disorders are frequent long-term consequences of injury but have insufficient treatment options. Therefore, this section will highlight the idea that deficits in affective behavior following TBI, and specifically mild TBI, may depend heavily on neuronal projections and neurochemicals originating within the amygdala (Han et al., 2015; Horn et al., 2016; Ratliff et al., 2019; Beitchman et al., 2020). The function, structure, and connectivity of the amygdala is disrupted following TBI suggesting that this region may be a source of behavioral impairment. For example, hyperactivity of the amygdala is suggested to be involved in the manifestation of affective disorders in brain-injured patients (Han et al., 2015). Clinical and preclinical neurobiological studies have supported the idea that the amygdala, a major component of the limbic system, may be a hub for regulating affective behaviors (Phelps and Ledoux, 2005; Etkin et al., 2010; Pessoa, 2010; Calhoon and Tye, 2015; Korgaonkar et al., 2019). The amygdala sends projections to multiple brain regions thereby involving it in the various circuits responsible for the expression of affective behaviors and is also home to a diverse array of neurotransmitters and neuropeptides which boosts its potential to mediate impairments in affective behaviors (Phelps and Ledoux, 2005; Etkin et al., 2010; Pessoa, 2010; Felix-Ortiz and Tye, 2014; Calhoon and Tye, 2015; McGarry and Carter, 2016; Paretkar and Dimitrov, 2018; Korgaonkar et al., 2019).

### Anatomic Pathways: The Basolateral and Central Amygdala

The BLA and CeA are the two primary subregions of the amygdala widely known for their involvement in depression and fear and anxiety, respectively. Their projections to various brain regions aid in the regulation of these disorders, in that disruption of these pathways can lead to disorder expression (Kim and Jung, 2006; Calhoon and Tye, 2015; Leal et al., 2017). This section will examine how the manifestation of depression, anxiety, or fear can occur through divergent circuitry arising from the various subregions of the amygdala.

Disruptions in the BLA-derived circuitry drive the expression of both cognitive and emotional aspects of depression-like behaviors. Preclinical studies show that the BLA exhibits neuronal hypertrophy in response to TBI (Hoffman et al., 2017) and increased dendritic branching following mild blast TBI (Ratliff et al., 2019). Animal models of depression exhibit impaired processing through the expression of anhedonia, despair, and prefrontal and hippocampal-dependent cognitive deficits (Paul et al., 2005; Der-Avakian and Markou, 2012). The BLA is also the main input nucleus receiving sensory information from the thalamus which is imbued with emotional value and associations are made between neural stimuli and outcomes of positive or negative valence (Janak and Tye, 2015). Cognitive components of depression are driven by the BLA coordinating emotional learning and memory via projections to the prefrontal cortex and the ventral hippocampus (McGarry and Carter, 2016; Leal et al., 2017). Disruptions in these pathways in humans lead to impaired emotional memory, creating a bias toward negative thoughts (Leal et al., 2017). Activation of BLA neurons following mild TBI in animals potentiates glutamatergic targets leading to excitotoxicity in other brain regions including the hippocampus (Reger et al., 2012). The BLA also projects to the nucleus accumbens shell, where it inhibits dopamine release leading to low motivational states (McGarry and Carter, 2016). A decrease in dopamine release and reuptake in the nucleus accumbens has been reported in the chronic period following TBI in animals (Chen et al., 2017) which may underlie decreased motivation that leads to a depressive phenotype. Moreover, activation of the BLA during REM sleep, which is reduced in brain-injured patients, is critical for sleep-dependent emotional processing (Mcgaugh, 2004; Mantua et al., 2017; Clark et al., 2020). When this processing is impaired, there is an increase in arousal to negative words and experiences seen in patients (Liu et al., 2012). Collectively, these data provide a basis for the BLA, via its connections to the prefrontal cortex, hippocampus, and nucleus accumbens to regulate the expression of post-traumatic depression.

The BLA also aids in recruiting CeA neurons in response to threatening cues (Calhoon and Tye, 2015). Brain imaging studies in rodents found significant alterations in the CeA following single and repetitive mild TBI which corresponded to impairments in affective behavior (Kulkarni et al., 2019). The coordinated activity of the CeA, the bed nucleus of the stria terminalis (BNST), the ventral hippocampus, and the prefrontal cortex is required for the interpretation of stimuli as threatening to produce an anxiety-like response. Threatening cues induce anxiogenic behavior because of increased activation of the CeA and the BNST, whereas hyperexcitation within the CeA promotes arousal and hypervigilance and leads to the association of non-threatening factors with potential danger (Calhoon and Tye, 2015). Information regarding potential threats flows forward from the CeA to the BNST, then to the hippocampus and prefrontal cortex, and back from the prefrontal cortex and the hippocampus to the CeA (Calhoon and Tye, 2015); dysfunction in the hippocampus and the prefrontal cortex also contributes to anxiety-like behaviors (Cominski et al., 2014). Fear and anxiety-provoking circuits share numerous similarities, and contextual fear conditioning requires strong input from the hippocampus to relate contextual cues to an aversive stimulus (Kim and Jung, 2006; Calhoon and Tye, 2015). Moreover, medial septum cholinergic inputs into the hippocampus are needed for appropriate processing of contextual cues as background information (Calhoon and Tye, 2015; Staib et al., 2018). The CeA also projects to the paraventricular nucleus of the hypothalamus (Beaulieu et al., 1986) and hyperactivity in the CeA leads to disruption of the hypothalamic-pituitary-adrenal (HPA) axis and anxiety-like behaviors and alterations in the natural fear response (Flandreau et al., 2012; Calhoon and Tye, 2015). In animals, TBI induces disruption in HPA circuitry resulting in compromised emotional regulation and, specifically, anxiety (Tapp et al., 2019). These data suggest that the CeA may be a likely nucleus mediating post-traumatic anxiety and fear behaviors.

### Neurochemical Pathways: γ-Amino Butyric Acid and CRF

Whereas different subregions of the amygdala may be more responsible for the expression of one affective disorder over the other, neurochemical transmission within the amygdala may contribute to the comorbid expression of depression, anxiety, and fear-related behaviors. These neurochemicals consist of the primary excitatory neurotransmitter glutamate, the primary inhibitory neurotransmitter γ-amino butyric acid (GABA), and CRF, the peptide hormone largely known to be involved in the stress response. Alterations in the concentration or activity of these neurochemicals have been linked to the manifestation of all three affective behavioral disorders, suggesting that they serve as points of integration of mechanisms underlying the convergence of depression, anxiety and fear.

Disruptions in the concentrations of GABA and glutamate are associated with deficits in affective behaviors (Calhoon and Tye, 2015; Jie et al., 2018). Activation of GABAergic neurons within the amygdala prevents the propagation of excitatory input to downstream regions and keeps anxiety responses in check (Calhoon and Tye, 2015). Either due to reduced GABA transmission or increased activity within the BLA following mild TBI, potentiation of glutamatergic activity may be associated with fear-related behaviors (Reger et al., 2012; McGuire et al., 2018). Anxiety-like behaviors following TBI in animals has been associated with decreased GAD expression in the BLA (Popovitz et al., 2019) or decreased evoked glutamate release and slower glutamate clearance within the CeA (Beitchman et al., 2020). Depression also manifests through imbalances in glutamate and GABA concentrations (Luscher et al., 2011; Luscher and Fuchs, 2015; Duman et al., 2019). For example, a postmortem study showed decreased levels of GABA expression in the BLA of patients with major depression disorder (Douillard-Guilloux et al., 2017). Thus, overall, reductions in GABA levels and excitotoxicity of glutamate, specifically within the amygdala, are major contributors to all three affective disorders, serving as a potential point of mechanistic convergence.

The expression of CRF is increased in the amygdala following brain injury (Narla et al., 2019; Tapp et al., 2019) and blockade of CRFR1 receptor 1 within the HPA axis attenuates post-traumatic stress and anxiety-like behaviors (Kosari-Nasab et al., 2019). The activity of CRF in extrahypothalamic regions contributes to depression, anxiety, and fear-related behaviors (Binder and Nemeroff, 2009; Sanford et al., 2017; Paretkar and Dimitrov, 2018). The CeA contains CRF-positive neurons (Rodaros et al., 2007), whereas the BLA contains numerous CRF receptor-positive projection neurons (Roozendaal et al., 2002). Increased CRF in the BLA can impair memory consolidation (Narla et al., 2019), whereas increased CRF in the nucleus accumbens leads to depressive behaviors likely by modulating extracellular acetylcholine (Chen et al., 2012); CRF receptor 1 antagonists exhibit antidepressant activity (Overstreet and Griebel, 2004). Neurons within the CeA that express CRF have been investigated for their role in perpetuating anxiety behaviors following chronic stress (Paretkar and Dimitrov, 2018; Hupalo et al., 2019) and promote fear learning by regulating acquisition recall (Sanford et al., 2017). Norepinephrine can promote GABA release from neurons thereby enhancing GABA-mediated inhibition of CRF neuronal activity (Levy and Tasker, 2012); norepinephrine is reduced following TBI suggesting that post-traumatic affective behaviors may be linked to CRF activity (McGuire et al., 2018). Together, these data suggest that hyperactivity of CRF neurons within the amygdala following injury may be a viable mechanistic underpinning of all three affective disorders.

### Integration of Amygdala Structure and Function

In psychology, emotional processing describes the way in which an individual successfully responds to emotional stimuli or stressful events, and impairment in processing can lead to signs of depression, anxiety, and fear (Rachman, 1980) which have a high incidence of comorbidity in a subset of brain-injured patients (Jorge et al., 2004; Ellis et al., 2015; Mohammad Farris Iman Leong Bin Abdullah et al., 2018; Teymoori et al., 2020). The amygdala is well-known for its involvement in emotional processing based on observations of structural and functional alterations in patients that develop affective disorders such as depression and fear (Belujon and Grace, 2011; Douillard-Guilloux et al., 2017). Thus, a decrease in the number of somatostatin-labeled neurons within the BLA of the amygdala was observed in patients with major depression (Douillard-Guilloux et al., 2017). Additionally, in patients with generalized anxiety disorder, functional MRI during an emotional conflict task revealed greater activation in the amygdala (Etkin et al., 2010). Similarly, patients with PTSD exhibited an increase in amygdala activation detected using either positron emission tomography (Bremner et al., 2005) or blood oxygen level dependent functional MRI (Protopopescu et al., 2005), particularly when exposed to negative stimuli. The subregions of the amygdala, specifically the BLA and the CeA, may be the points of divergence in that impairment in each subregion is associated with a specific deficit. Thus, disruption in the BLA can result in the expression of depressive-like behaviors (Douillard-Guilloux et al., 2017), whereas impairment to the CeA can result in anxiety or fear-related behaviors (Calhoon and Tye, 2015). Because it is structurally and functionally impaired following mild TBI, the amygdala has been highlighted as critical to the development of post-traumatic deficits in affective behaviors. The true integration of these disorders lies in the connectivity between, and the neurochemical interactions within these two subregions of the amygdala. Therefore, impairment in the circuitry of BLA and CeA as well as disruptions in GABA or CRF concentrations post-injury have the potential to lead to comorbid expression of depression, anxiety, and fear-related behaviors. In these instances, the behavioral consequence exhibited would be one of a collective impairment in emotional processing.

## Future Directions

Mild TBI is a public health concern that greatly impacts the lives of adolescents who suffer from sports-related concussion and young adults who experience mild blast TBI. A primary consequence of mild TBI is the development of long-term deficits in affective behaviors which have been reported in both humans and animals. It is important to note that the comorbidity of depression, anxiety, and fear-related behaviors are only observed in a subset of patients, and the appearance of these behaviors in preclinical models depends on the impact site and frequency of the repetitive injury. These behaviors have been largely understudied and there are few treatments in use currently that attenuate post-traumatic depression, anxiety, and fear/PTSD. Future clinical and preclinical studies need to examine the impairment in amygdala circuitry and neurotransmission following mild TBI. One of the major structural impairments following mild TBI is axonal injury and brain imaging must examine which projections to and from the different subregions of the amygdala are disrupted. In addition, alterations in the expression and activity of specific neurotransmitters must also be evaluated for the behavioral deficit being studied. Treatment paradigms therefore need to examine structural and functional alterations in the amygdala concomitantly with disruption in specific neurotransmitters facilitating an integrated approach, potentially ameliorating depression, anxiety, and fear-related behaviors together.

## Author Contributions

TM, JB, and RR wrote and edited the manuscript. All authors contributed to the article and approved the submitted version.

## Conflict of Interest

The authors declare that the research was conducted in the absence of any commercial or financial relationships that could be construed as a potential conflict of interest.

## References

[B1] Almeida-Suhett C. P. Prager E. M. Pidoplichko V. Figueiredo T. H. Marini A. M. Li Z. (2014). Reduced GABAergic inhibition in the basolateral amygdala and the development of anxiety-like behaviors after mild traumatic brain injury. *PLoS One* 9:e102627. 10.1371/journal.pone.0102627 25047645 PMC4105413

[B2] Baldassarre M. Smith B. Harp J. Herrold A. High W. M. Babcock-Parziale J. (2015). Pape TL-B: exploring the relationship between mild traumatic brain injury exposure and the presence and severity of postconcussive symptoms among veterans deployed to iraq and afghanistan. *PM R* 7 845–858. 10.1016/j.pmrj.2015.03.003 25758529

[B3] Baldwin G. T. Breiding M. J. Comstock R. D. (2018). Epidemiology of sports concussion in the United States. *Sports Neurol. Handb. Clin. Neurol.* 158 63–74. 10.1016/b978-0-444-63954-7.00007-0 30482376

[B4] Bazarian J. J. Blyth B. Mookerjee S. He H. Mcdermott M. P. (2010). Sex differences in outcome after mild traumatic brain injury. *J. Neurotrauma* 27 527–539. 10.1089/neu.2009.1068 19938945 PMC2867588

[B5] Beaulieu S. Paolo T. D. Barden N. (1986). Control of ACTH secretion by the central nucleus of the amygdala: implication of the serotoninergic system and its relevance to the glucocorticoid delayed negative feedback mechanism. *Neuroendocrinology* 44 247–254. 10.1159/000124652 3025760

[B6] Beitchman J. A. Griffiths D. R. Hur Y. Ogle S. B. Bromberg C. E. Morrison H. W. (2020). Experimental traumatic brain injury induces chronic glutamatergic dysfunction in amygdala circuitry known to regulate anxiety-like behavior. *Front. Neurosci.* 13:1434. 10.3389/fnins.2019.01434 PMC6985437 32038140

[B7] Belujon P. Grace A. A. (2011). Hippocampus, amygdala, and stress: interacting systems that affect susceptibility to addiction. *Ann. N. Y. Acad. Sci.* 1216 114–121. 10.1111/j.1749-6632.2010.05896.x 21272015 PMC3141575

[B8] Bhattrai A. Irimia A. Horn J. D. V. (2019). Neuroimaging of traumatic brain injury in military personnel: an overview. *J. Clin. Neurosci.* 70 1–10. 10.1016/j.jocn.2019.07.001 31331746 PMC6861663

[B9] Binder E. B. Nemeroff C. B. (2009). The CRF system, stress, depression and anxiety—insights from human genetic studies. *Mol. Psychiatry* 15 574–588. 10.1038/mp.2009.141 20010888 PMC3666571

[B10] Blaze J. Choi I. Wang Z. Umali M. Mendelev N. Tschiffely A. E. (2020). Blast-related mild TBI alters anxiety-like behavior and transcriptional signatures in the rat amygdala. *Front. Behav. Neurosci.* 14:160. 10.3389/fnbeh.2020.00160 PMC7604767 33192359

[B11] Bodnar C. N. Roberts K. N. Higgins E. K. Bachstetter A. D. (2019). A systematic review of closed head injury models of mild traumatic brain injury in mice and rats. *J. Neurotrauma* 36 1683–1706. 10.1089/neu.2018.6127 30661454 PMC6555186

[B12] Brassil H. E. Salvatore A. P. (2018). The frequency of post-traumatic stress disorder symptoms in athletes with and without sports related concussion. *Clin. Transl. Med.* 7:25. 10.1186/s40169-018-0200-y PMC6056355 30039260

[B13] Bremner D. J. Vermetten E. Schmahl C. Vaccarino V. Vythilingam M. Afzal N. (2005). Positron emission tomographic imaging of neural correlates of a fear acquisition and extinction paradigm in women with children sexual-abuse-related post-traumatic stress disorder. *Psychol. Med.* 35 791–806. 10.1017/s0033291704003290 15997600 PMC3233760

[B14] Brewin C. R. Cloitre M. Hyland P. Shevlin M. Maercker A. Bryant R. A. (2017). A review of current evidence regarding the ICD-11 proposals for diagnosing PTSD and complex PTSD. *Clin. Psychol. Rev.* 58 1–15. 10.1016/j.cpr.2017.09.001 29029837

[B15] Broshek D. K. Kaushik T. Freeman J. R. Erlanger D. Webbe F. Barth J. T. (2005). Sex differences in outcome following sports-related concussion. *J. Neurosurg.* 102 856–863. 10.3171/jns.2005.102.5.0856 15926710

[B16] Bunt S. C. Didehbani N. Tarkenton T. Rossetti H. Hicks C. Vargas B. (2020). Sex differences and reporting of SCAT-5 concussion symptoms in adolescent athletes. *Clin. J. Sport Med.* 10.1097/JSM.0000000000000788 Online ahead of print. 31985537

[B17] Calhoon G. G. Tye K. M. (2015). Resolving the neural circuits of anxiety. *Nat. Neurosci.* 18 1394–1404. 10.1038/nn.4101 26404714 PMC7575249

[B18] Can A. Dao D. T. Arad M. Terrillion C. E. Piantadosi S. C. Gould T. D. (2011). The mouse forced swim test. *J. Vis. Exp.* 59:e3638. 10.3791/3638 PMC3353513 22314943

[B19] Can A. Dao D. T. Terrillion C. E. Piantadosi S. C. Shambhu B. Gould T. D. (2012). The tail suspension test. *J. Vis. Exp.* 59:3769. 10.3791/3769 22315011 PMC3353516

[B20] Chen Y.-H. Huang E. Y.-K. Kuo T.-T. Hoffer B. J. Miller J. Chou Y.-C. (2017). Dopamine release in the nucleus accumbens is altered following traumatic brain injury. *Neuroscience* 348 180–190. 10.1016/j.neuroscience.2017.02.001 28196657

[B21] Chen Y.-W. Rada P. Bützler B. Leibowitz S. Hoebel B. (2012). Corticotropin-releasing factor in the nucleus accumbens shell induces swim depression, anxiety, and anhedonia along with changes in local dopamine/acetylcholine balance. *Neuroscience* 206 155–166. 22245501 10.1016/j.neuroscience.2011.12.009

[B22] Cheng H. C. Martens K. M. Bashie A. Cheung H. Stukas S. Gibbs E. (2019). CHIMERA repetitive mild traumatic brain injury induces chronic behavioral and neuropathological phenotypes in wild-type and APP/PS1 mice. *Alzheimers Res. Ther.* 11:6. 10.1186/s13195-018-0461-0 PMC6330571 30636629

[B23] Cheng J. S. Craft R. Yu G.-Q. Ho K. Wang X. Mohan G. (2014). Tau reduction diminishes spatial learning and memory deficits after mild repetitive traumatic brain injury in mice. *PLoS One* 9:e115765. 10.1371/journal.pone.0115765 25551452 PMC4281043

[B24] Chong C. D. Schwedt T. J. (2018). Research imaging of brain structure and function after concussion. *Headache J. Head Face Pain* 58 827–835. 10.1111/head.13269 29476532

[B25] Clark J. W. Hoyer D. Cain S. W. Phillips A. Drummond S. P. Jacobson L. H. (2020). Circadian disruption impairs fear extinction and memory of conditioned safety in mice. *Behav. Brain Res.* 393:112788. 10.1016/j.bbr.2020.112788 32598999

[B26] Cominski T. P. Jiao X. Catuzzi J. E. Stewart A. L. Pang K. C. H. (2014). The role of the hippocampus in avoidance learning and anxiety vulnerability. *Front. Behav. Neurosci.* 8:273. 10.3389/fnbeh.2014.00273 PMC4125878 25152721

[B27] Cooper D. B. Bunner A. E. Kennedy J. E. Balldin V. Tate D. F. Eapen B. C. (2015). Treatment of persistent post-concussive symptoms after mild traumatic brain injury: a systematic review of cognitive rehabilitation and behavioral health interventions in military service members and veterans. *Brain Imaging Behav.* 9 403–420. 10.1007/s11682-015-9440-2 26330376

[B28] Coronado V. G. Xu L. Basavaraju S. V. McGuire L. C. Wald M. M. Faul M. D. (2011). Surveillance for traumatic brain injury-related deaths—United States. *MMWR Surveill. Summ.* 60 1–32. 21544045

[B29] Cryan J. F. Markou A. Lucki I. (2002). Assessing antidepressant activity in rodents: recent developments and future needs. *Trends Pharmacol. Sci.* 23 238–245. 10.1016/s0165-6147(02)02017-5 12008002

[B30] Der-Avakian A. Markou A. (2012). The neurobiology of anhedonia and other reward-related deficits. *Trends Neurosci.* 35 68–77. 10.1016/j.tins.2011.11.005 22177980 PMC3253139

[B31] Douillard-Guilloux G. Lewis D. Seney M. L. Sibille E. (2017). Decrease in somatostatin-positive cell density in the amygdala of females with major depression. *Depress. Anxiety* 34 68–78. 10.1002/da.22549 27557481 PMC5222785

[B32] Duman R. S. Sanacora G. Krystal J. H. (2019). Altered connectivity in depression: GABA and glutamate neurotransmitter deficits and reversal by novel treatments. *Neuron* 102 75–90. 10.1016/j.neuron.2019.03.013 30946828 PMC6450409

[B33] Eagle A. Mazei-Robison M. Robison A. (2016). Sucrose preference test to measure stress-induced anhedonia. *Bio Protocol.* 6 1686–1698.

[B34] Ellis M. J. Ritchie L. J. Koltek M. Hosain S. Cordingley D. Chu S. (2015). Psychiatric outcomes after pediatric sports-related concussion. *J. Neurosurg. Pediatr.* 16 709–718. 10.3171/2015.5.peds15220 26359916

[B35] Etkin A. Prater K. E. Hoeft F. Menon V. Schatzberg A. F. (2010). Failure of anterior cingulate activation and connectivity with the amygdala during implicit regulation of emotional processing in generalized anxiety disorder. *Am. J. Psychiatry* 167 545–554. 10.1176/appi.ajp.2009.09070931 20123913 PMC4367202

[B36] Fann J. R. Bombardier C. H. Vannoy S. Dyer J. Ludman E. Dikmen S. (2015). Telephone and in-person cognitive behavioral therapy for major depression after traumatic brain injury: a randomized controlled trial. *J. Neurotrauma* 32 45–57. 10.1089/neu.2014.3423 25072405 PMC4273196

[B37] Felix-Ortiz A. C. Tye K. M. (2014). Amygdala inputs to the ventral hippocampus bidirectionally modulate social behavior. *J. Neurosci.* 34 586–595. 10.1523/jneurosci.4257-13.2014 24403157 PMC3870937

[B38] Flandreau E. I. Ressler K. J. Owens M. J. Nemeroff C. B. (2012). Chronic overexpression of corticotropin-releasing factor from the central amygdala produces HPA axis hyperactivity and behavioral anxiety associated with gene-expression changes in the hippocampus and paraventricular nucleus of the hypothalamus. *Psychoneuroendocrinology* 37 27–38. 10.1016/j.psyneuen.2011.04.014 21616602 PMC3164918

[B39] Flower O. Hellings S. (2012). Sedation in traumatic brain injury. *Emerg. Med. Intl.* 2012 1–11. 10.1155/2012/637171 PMC3461283 23050154

[B40] Fortress A. M. Avcu P. Wagner A. K. Dixon C. E. Pang K. C. (2019). Experimental traumatic brain injury results in estrous cycle disruption, neurobehavioral deficits, and impaired GSK3β/β-catenin signaling in female rats. *Exp. Neurol.* 315 42–51. 10.1016/j.expneurol.2019.01.017 30710530

[B41] Fox L. C. Davies D. R. Scholl J. L. Watt M. J. Forster G. L. (2016). Differential effects of glucocorticoid and mineralocorticoid antagonism on anxiety behavior in mild traumatic brain injury. *Behav. Brain Res.* 312 362–365. 10.1016/j.bbr.2016.06.048 27363926

[B42] Gao Y. Tuvblad C. Schell A. Baker L. Raine A. (2014). Skin conductance fear conditioning impairments and aggression: a longitudinal study. *Psychophysiology* 52 288–295. 10.1111/psyp.12322 25174802 PMC4304981

[B43] Gardner R. C. Yaffe K. (2016). Epidemiology of mild traumatic brain injury and neurodegenerative disease. *Mol. Cell. Neurosci.* 66 75–80. 10.1016/j.mcn.2015.03.001 25748121 PMC4461453

[B44] Giguère F. L. Frasnelli A. Guise Éd Frasnelli J. (2019). Olfactory, cognitive and affective dysfunction assessed 24 hours and one year after a mild traumatic brain injury (mTBI). *Brain Injury* 33 1184–1193. 10.1080/02699052.2019.1631486 31223039

[B45] Glenn D. E. Acheson D. T. Geyer M. A. Nievergelt C. M. Baker D. G. Risbrough V. B. (2017). Fear learning alterations after traumatic brain injury and their role in development of posttraumatic stress symptoms. *Depress. Anxiety* 34 723–733. 10.1002/da.22642 28489272 PMC12128915

[B46] Grima N. A. Rajaratnam S. M. W. Mansfield D. Sletten T. L. Spitz G. Ponsford J. L. (2018). Efficacy of melatonin for sleep disturbance following traumatic brain injury: a randomised controlled trial. *BMC Med.* 16:8. 10.1186/s12916-017-0995-1 PMC5774131 29347988

[B47] Gupta A. Summerville G. Senter C. (2019). Treatment of acute sports-related concussion. *Curr. Rev. Musculoskeletal Med.* 12 117–123. 10.1007/s12178-019-09545-7 PMC6542872 30887284

[B48] Han K. Chapman S. B. Krawczyk D. C. (2015). Altered amygdala connectivity in individuals with chronic traumatic brain injury and comorbid depressive symptoms. *Front. Neurol.* 6:231. 10.3389/fneur.2015.00231 PMC4631949 26581959

[B49] Heldt S. A. Elberger A. J. Deng Y. Guley N. H. Del Mar N. Rogers J. (2014). A novel closed-head model of mild traumatic brain injury caused by primary overpressure blast to the cranium produces sustained emotional deficits in mice. *Front. Neurol.* 6:2. 10.3389/fneur.2014.00002 PMC3898331 24478749

[B50] Herrera J. J. Bockhorst K. Kondraganti S. Stertz L. Quevedo J. Narayana P. A. (2017). Acute white matter tract damage after frontal mild traumatic brain injury. *J. Neurotrauma* 34 291–299. 10.1089/neu.2016.4407 27138134 PMC5220577

[B51] Hoffman A. N. Lam J. Hovda D. A. Giza C. C. Fanselow M. S. (2019). Sensory sensitivity as a link between concussive traumatic brain injury and PTSD. *Sci. Rep.* 9:13841. 10.1038/s41598-019-50312-y PMC6761112 31554865

[B52] Hoffman A. N. Paode P. R. May H. G. Ortiz J. B. Kemmou S. Lifshitz J. (2017). Early and persistent dendritic hypertrophy in the basolateral amygdala following experimental diffuse traumatic brain injury. *J. Neurotrauma* 34 213–219. 10.1089/neu.2015.4339 27306143 PMC5198057

[B53] Hoogenboom W. S. Rubin T. G. Ye K. Cui M. Branch K. C. Liu J. (2019). Diffusion tensor imaging of the evolving response to mild traumatic brain injury in rats. *J. Exp. Neurosci.* 13:1179069519858627. 10.1177/1179069519858627 PMC6613065 31308735

[B54] Horn H. J. V. D. Liemburg E. J. Scheenen M. E. Koning M. E. D. Marsman J.-B. C. Spikman J. M. (2016). Brain network dysregulation, emotion, and complaints after mild traumatic brain injury. *Hum. Brain Mapp.* 37 1645–1654. 10.1002/hbm.23126 26846195 PMC6867381

[B55] Hupalo S. Bryce C. A. Bangasser D. A. Berridge C. W. Valentino R. J. Floresco S. B. (2019). Corticotropin-releasing factor (CRF) circuit modulation of cognition and motivation. *Neurosci. Biobehav. Rev.* 103 50–59. 10.1016/j.neubiorev.2019.06.010 31212019 PMC6692202

[B56] Janak P. H. Tye K. M. (2015). From circuits to behaviour in the amygdala. *Nature* 517 284–292. 10.1038/nature14188 25592533 PMC4565157

[B57] Jie F. Yin G. Yang W. Yang M. Gao S. Lv J. (2018). Stress in regulation of GABA amygdala system and relevance to neuropsychiatric diseases. *Front. Neurosci.* 12:562. 10.3389/fnins.2018.00562 PMC6103381 30154693

[B58] Jorge R. E. Robinson R. G. Moser D. Tateno A. Crespo-Facorro B. Arndt S. (2004). Major depression following traumatic brain injury. *Arch. Gen. Psychiatry* 61:42. 10.1001/archpsyc.61.1.42 14706943

[B59] Kay T. Harrington D. E. Adams R. Anderson T. Berrol S. Cicerone K. (1993). Definition of mild traumatic brain injury. *J. Head Trauma Rehabil.* 8 86–87.

[B60] Kikinis Z. Muehlmann M. Pasternak O. Peled S. Kulkarni P. Ferris C. (2017). Diffusion imaging of mild traumatic brain injury in the impact accelerated rodent model: a pilot study. *Brain Injury* 31 1376–1381. 10.1080/02699052.2017.1318450 28627942 PMC5896003

[B61] Kim J. J. Jung M. W. (2006). Neural circuits and mechanisms involved in pavlovian fear conditioning: a critical review. *Neurosci. Biobehav. Rev.* 30 188–202. 10.1016/j.neubiorev.2005.06.005 16120461 PMC4342048

[B62] Kim M. J. Loucks R. A. Palmer A. L. Brown A. C. Solomon K. M. Marchante A. N. (2011). The structural and functional connectivity of the amygdala: from normal emotion to pathological anxiety. *Behav. Brain Res.* 223 403–410. 10.1016/j.bbr.2011.04.025 21536077 PMC3119771

[B63] Korgaonkar M. S. Erlinger M. Breukelaar I. A. Boyce P. Hazell P. Antees C. (2019). Amygdala activation and connectivity to emotional processing distinguishes asymptomatic patients with bipolar disorders and unipolar depression. *Biol. Psychiatry Cogn. Neurosci. Neuroimaging* 4 361–370. 10.1016/j.bpsc.2018.08.012 30343134

[B64] Kosari-Nasab M. Sadeghi T. Bashiri H. Shokouhi G. Salari A.-A. (2019). The blockade of corticotropin-releasing factor 1 receptor attenuates anxiety-related symptoms and hypothalamus–pituitary–adrenal axis reactivity in mice with mild traumatic brain injury. *Behav. Pharmacol.* 30 220–228. 10.1097/fbp.0000000000000450 30883392

[B65] Kraus C. Castrén E. Kasper S. Lanzenberger R. (2017). Serotonin and neuroplasticity – links between molecular, functional and structural pathophysiology in depression. *Neurosci. Biobehav. Rev.* 77 317–326. 10.1016/j.neubiorev.2017.03.007 28342763

[B66] Kuehn R. Simard P. F. Driscoll I. Keledjian K. Ivanova S. Tosun C. (2011). Rodent model of direct cranial blast injury. *J. Neurotrauma* 28 2155–2169. 10.1089/neu.2010.1532 21639724

[B67] Kulkarni P. Morrison T. R. Cai X. Iriah S. Simon N. Sabrick J. (2019). Neuroradiological changes following single or repetitive mild TBI. *Front. Syst. Neurosci.* 13:34. 10.3389/fnsys.2019.00034 PMC6688741 31427931

[B68] Lange R. T. Brickell T. A. Bailie J. M. Tulsky D. S. French L. M. (2016). Clinical utility and psychometric properties of the traumatic brain injury quality of life scale (TBI-QOL) in US military service members. *J. Head Trauma Rehabil.* 31 62–78. 10.1097/htr.0000000000000149 26716697

[B69] Lavoie S. Sechrist S. Quach N. Ehsanian R. Duong T. Gotlib I. H. (2017). Depression in men and women one year following traumatic brain injury (TBI): a tbi model systems study. *Front. Psychol.* 8:634. 10.3389/fpsyg.2017.00634 PMC5418333 28529492

[B70] Leal S. L. Noche J. A. Murray E. A. Yassa M. A. (2017). Disruption of amygdala-entorhinal-hippocampal network in late-life depression. *Hippocampus* 27 464–476. 10.1002/hipo.22705 28085210 PMC5858586

[B71] Levy B. H. Tasker J. G. (2012). Synaptic regulation of the hypothalamic–pituitary–adrenal axis and its modulation by glucocorticoids and stress. *Front. Cell. Neurosci.* 6:24. 10.3389/fncel.2012.00024 PMC3349941 22593735

[B72] Lim S.-W. Sung K.-C. Shiue Y.-L. Wang C.-C. Chio C.-C. Kuo J.-R. (2017). Hyperbaric oxygen effects on depression-like behavior and neuroinflammation in traumatic brain injury rats. *World Neurosurg.* 100 128–137. 10.1016/j.wneu.2016.12.118 28065873

[B73] Liu M.-Y. Yin C.-Y. Zhu L.-J. Zhu X.-H. Xu C. Luo C.-X. (2018). Sucrose preference test for measurement of stress-induced anhedonia in mice. *Nat. Protoc.* 13 1686–1698. 10.1038/s41596-018-0011-z 29988104

[B74] Liu W.-H. Wang L.-Z. Zhao S.-H. Ning Y.-P. Chan R. C. (2012). Anhedonia and emotional word memory in patients with depression. *Psychiatry Res.* 200 361–367. 10.1016/j.psychres.2012.07.025 22910474

[B75] Luscher B. Fuchs T. (2015). “GABAergic control of depression-related brain states,” in *Diversity and Functions of GABA Receptors: a Tribute to Hanns Möhler, Part b Advances in Pharmacology*, ed. Rudolph U. (Cambridge, MA: Academic Press). 10.1016/bs.apha.2014.11.003 PMC4784429 25637439

[B76] Luscher B. Shen Q. Sahir N. (2011). The GABAergic deficit hypothesis of major depressive disorder. *Mol. Psychiatry* 16 383–406. 10.1038/mp.2010.120 21079608 PMC3412149

[B77] Ma X. Aravind A. Pfister B. J. Chandra N. Haorah J. (2019). Animal models of traumatic brain injury and assessment of injury severity. *Mol. Neurobiol.* 56 5332–5345. 10.1007/s12035-018-1454-5 30603958

[B78] Maeng L. Y. Milad M. R. (2015). Sex differences in anxiety disorders: interactions between fear, stress, and gonadal hormones. *Horm. Behav.* 76 106–117. 10.1016/j.yhbeh.2015.04.002 25888456 PMC4823998

[B79] Malkesman O. Tucker L. B. Ozl J. Mccabe J. T. (2013). Traumatic brain injury – modeling neuropsychiatric symptoms in rodents. *Front. Neurol.* 4:157. 10.3389/fneur.2013.00157 PMC3791674 24109476

[B80] Manley G. Gardner A. J. Schneider K. J. Guskiewicz K. M. Bailes J. Cantu R. C. (2017). A systematic review of potential long-term effects of sport-related concussion. *Br. J. Sports Med.* 51 969–977. 10.1136/bjsports-2017-097791 28455362 PMC5466926

[B81] Mantua J. Henry O. S. Garskovas N. F. Spencer R. M. (2017). Mild traumatic brain injury chronically impairs sleep- and wake-dependent emotional processing. *Sleep* 40:zsx062. 10.1093/sleep/zsx062 PMC5806572 28460124

[B82] McGarry L. M. Carter A. G. (2016). Inhibitory gating of basolateral amygdala inputs to the prefrontal cortex. *J. Neurosci.* 36 9391–9406. 10.1523/jneurosci.0874-16.2016 27605614 PMC5013187

[B83] Mcgaugh J. L. (2004). The amygdala modulates the consolidation of memories of emotionally arousing experiences. *Annu. Rev. Neurosci.* 27 1–28. 10.1146/annurev.neuro.27.070203.144157 15217324

[B84] McGuire J. L. Ngwenya L. B. Mccullumsmith R. E. (2018). Neurotransmitter changes after traumatic brain injury: an update for new treatment strategies. *Mol. Psychiatry* 24 995–1012. 10.1038/s41380-018-0239-6 30214042

[B85] McKee A. C. Robinson M. E. (2014). Military-related traumatic brain injury and neurodegeneration. *Alzheimer’s Dement.* 10 242–253. 10.1016/j.jalz.2014.04.003 PMC4255273 24924675

[B86] McMahon P. J. Hricik A. Yue J. K. Puccio A. M. Inoue T. Lingsma H. F. (2014). Symptomatology and functional outcome in mild traumatic brain injury: results from the prospective TRACK-TBI study. *J. Neurotrauma* 31 26–33. 10.1089/neu.2013.2984 23952719 PMC3880097

[B87] Mierzwa A. J. Marion C. M. Sullivan G. M. McDaniel D. P. Armstrong R. C. (2015). Components of myelin damage and repair in the progression of white matter pathology after mild traumatic brain injury. *J. Neuropathol. Exp. Neurol.* 74 218–232. 10.1097/nen.0000000000000165 25668562 PMC4327393

[B88] Mihalik J. P. Ondrak K. S. Guskiewicz K. M. Mcmurray R. G. (2009). The effects of menstrual cycle phase on clinical measures of concussion in healthy college-aged females. *J. Sci. Med. Sport* 12 383–387. 10.1016/j.jsams.2008.05.003 18771954

[B89] Mohammad Farris Iman Leong Bin Abdullah Ng Y. P. Sidi H. B. (2018). Depression and anxiety among traumatic brain injury patients in Malaysia. *Asian J. Psychiatry* 37 67–70. 10.1016/j.ajp.2018.08.017 30144779

[B90] Mustafi S. M. Harezlak J. Koch K. M. Nencka A. S. Meier T. B. West J. D. (2018). Acute white-matter abnormalities in sports-related concussion: a diffusion tensor imaging study from the NCAA-DoD CARE consortium. *J. Neurotrauma* 22 2653–2664. 10.1089/neu.2017.5158 29065805 PMC6238613

[B91] Narla C. Jung P. S. Cruz F. B. Everest M. Martinez-Trujillo J. Poulter M. O. (2019). CRF mediates stress-induced pathophysiological high-frequency oscillations in traumatic brain injury. *eneuro* 6:ENEURO.0334-18. 10.1523/ENEURO.0334-18.2019 PMC6514440 31040158

[B92] Nolan A. Hennessy E. Krukowski K. Guglielmetti C. Chaumeil M. M. Sohal V. S. (2018). Repeated mild head injury leads to wide-ranging deficits in higher-order cognitive functions associated with the prefrontal cortex. *J. Neurotrauma* 35 2425–2434. 10.1089/neu.2018.5731 29732949 PMC6196749

[B93] Overstreet D. H. Griebel G. (2004). Antidepressant-like effects of CRF1 receptor antagonist SSR125543 in an animal model of depression. *Eur. J. Pharmacol.* 497 49–53. 10.1016/j.ejphar.2004.06.035 15321734

[B94] Palmer C. P. Metheny H. E. Elkind J. A. Cohen A. S. (2016). Diminshed amygdala activation and behavioral threat response following traumatic brain injury. *Exp. Neurol.* 277 215–226. 10.1016/j.expneurol.2016.01.004 26791254 PMC4761321

[B95] Paretkar T. Dimitrov E. (2018). The central amygdala corticotropin-releasing hormone (CRH) neurons modulation of anxiety-like behavior and hippocampus-dependent memory in mice. *Neuroscience* 390 187–197. 10.1016/j.neuroscience.2018.08.019 30170157 PMC6168391

[B96] Paul E. S. Harding E. J. Mendl M. (2005). Measuring emotional processes in animals: the utility of a cognitive approach. *Neurosci. Biobehav. Rev.* 29 469–491. 10.1016/j.neubiorev.2005.01.002 15820551

[B97] Perez-Garcia G. Gasperi R. D. Sosa M. A. G. Perez G. M. Otero-Pagan A. Tschiffely A. (2018). PTSD-related behavioral traits in a rat model of blast-induced mTBI are reversed by the mGluR2/3 receptor antagonist BCI-838. *eneuro* 5:ENEURO.357-17. 10.1523/ENEURO.0357-17.2018 PMC5790754 29387781

[B98] Pessoa L. (2010). Emotion and cognition and the amygdala: from “what is it?” to “whats to be done?”. *Neuropsychologia* 48 3416–3429. 10.1016/j.neuropsychologia.2010.06.038 20619280 PMC2949460

[B99] Petraglia A. L. Plog B. A. Dayawansa S. Chen M. Dashnaw M. L. Czerniecka K. (2014). The spectrum of neurobehavioral sequelae after repetitive mild traumatic brain injury: a novel mouse model of chronic traumatic encephalopathy. *J. Neurotrauma* 31 1211–1224. 10.1089/neu.2013.3255 24766454 PMC4082360

[B100] Phelps E. A. Ledoux J. E. (2005). Contributions of the amygdala to emotion processing: from animal models to human behavior. *Neuron* 48 175–187. 10.1016/j.neuron.2005.09.025 16242399

[B101] Plantier D. Luauté J. (2016). Drugs for behavior disorders after traumatic brain injury: systematic review and expert consensus leading to French recommendations for good practice. *Ann. Phys. Rehabil. Med.* 59 42–57. 10.1016/j.rehab.2015.10.003 26797170

[B102] Ponsford J. Lee N. K. Wong D. Mckay A. Haines K. Alway Y. (2015). Efficacy of motivational interviewing and cognitive behavioral therapy for anxiety and depression symptoms following traumatic brain injury. *Psychol. Med.* 46 1079–1090. 10.1017/s0033291715002640 26708017

[B103] Popovitz J. Mysore S. P. Adwanikar H. (2019). Long-term effects of traumatic brain injury on anxiety-like behaviors in mice: behavioral and neural correlates. *Front. Behav. Neurosci.* 13:6. 10.3389/fnbeh.2019.00006 PMC6351473 30728770

[B104] Prins M. Greco T. Alexander D. Giza C. C. (2013). The pathophysiology of traumatic brain injury at a glance. *Dis. Models Mech.* 6 1307–1315. 10.1242/dmm.011585 PMC3820255 24046353

[B105] Protopopescu X. Pan H. Tuescher O. Cloitre M. Goldstein M. Engelien W. (2005). Differential time courses and specificity of amygdala activity in posttraumatic stress disorder subject and normal control subjects. *Biol. Psychiatry* 57 464–473. 10.1016/j.biopsych.2004.12.026 15737660

[B106] Pryor J. Larson A. Debeliso M. (2016). The prevalence of depression and concussions in a sample of active north american semi-professional and professional football players. *J. Lifestyle Med.* 6 7–15. 10.15280/jlm.2016.6.1.7 27358835 PMC4915762

[B107] Rachman S. (1980). Emotional processing. *Behav. Res. Ther.* 18 51–60. 7369988 10.1016/0005-7967(80)90069-8

[B108] Rao V. Bertrand M. Rosenberg P. Makley M. Schretlen D. J. Brandt J. (2010). Predictors of new-onset depression after mild traumatic brain injury. *J. Neuropsychiatry Clin. Neurosci.* 22 100–104. 10.1176/jnp.2010.22.1.100 20160216 PMC2918274

[B109] Ratliff W. A. Mervis R. F. Citron B. A. Schwartz B. Rubovitch V. Schreiber S. (2019). Mild blast-related TBI in a mouse model alters amygdalar neurostructure and circuitry. *Exp. Neurol.* 315 9–14. 10.1016/j.expneurol.2019.01.020 30711646 PMC6622172

[B110] Reger M. L. Poulos A. M. Buen F. Giza C. C. Hovda D. A. Fanselow M. S. (2012). Concussive brain injury enhances fear learning and excitatory processes in the amygdala. *Biol. Psychiatry* 71 335–343. 10.1016/j.biopsych.2011.11.007 22169439 PMC3264758

[B111] Roberts A. L. Pascual-Leone A. Speizer F. E. Zafonte R. D. Baggish A. L. Taylor H. (2019). Exposure to American football and neuropsychiatric health in former national football league players: findings from the football players health study. *Am. J. Sports Med.* 47 2871–2880. 10.1177/0363546519868989 31468987 PMC7163246

[B112] Robinson S. Berglass J. B. Denson J. L. Berkner J. Anstine C. V. Winer J. L. (2017). Microstructural and microglial changes after repetitive mild traumatic brain injury in mice. *J. Neurosci. Res.* 95 1025–1035. 10.1002/jnr.23848 27452502 PMC5266745

[B113] Rodaros D. Caruana D. Amir S. Stewart J. (2007). Corticotropin-releasing factor projections from limbic forebrain and paraventricular nucleus of the hypothalamus to the region of the ventral tegmental area. *Neuroscience* 150 8–13. 10.1016/j.neuroscience.2007.09.043 17961928

[B114] Roozendaal B. Brunson K. L. Holloway B. L. Mcgaugh J. L. Baram T. Z. (2002). Involvement of stress-released corticotropin-releasing hormone in the basolateral amygdala in regulating memory consolidation. *Proc. Natl. Acad. Sci. U.S.A.* 99 13908–13913. 10.1073/pnas.212504599 12361983 PMC129796

[B115] Rowe R. K. Harrison J. L. Thomas T. C. Pauly J. R. Adelson D. P. Lifshitz J. (2014). Anesthetic and analgesics in experimental traumatic brain injury: selection based on experimental objectives. *Lab Anim.* 48 286–291. 10.1038/laban.257 23877609 PMC3876742

[B116] Ryan L. M. Warden D. L. (2003). Post concussion syndrome. *Int. Rev. Psychiatry* 15 310–316. 15276952 10.1080/09540260310001606692

[B117] Sanford C. A. Soden M. E. Baird M. A. Miller S. M. Schulkin J. Palmiter R. D. (2017). A central amygdala crf circuit facilitates learning about weak threats. *Neuron* 93 164–178. 10.1016/j.neuron.2016.11.034 28017470 PMC5217711

[B118] Schneiderman A. I. Braver E. R. Kang H. K. (2008). Understanding sequelae of injury mechanisms and mild traumatic brain injury incurred during the conflicts in iraq and afghanistan: persistent postconcussive symptoms and posttraumatic stress disorder. *Am. J. Epidemiol.* 167 1446–1452. 10.1093/aje/kwn068 18424429

[B119] Scholten A. C. Haagsma J. A. Cnossen M. C. Olff M. Beeck E. F. V. Polinder S. (2016). Prevalence of and risk factors for anxiety and depressive disorders after traumatic brain injury: a systematic review. *J. Neurotrauma* 33 1969–1994. 10.1089/neu.2015.4252 26729611

[B120] Schwenk T. L. Gorenflo D. W. Dopp R. R. Hipple E. (2007). Depression and pain in retired professional football players. *Med. Sci. Sports Exerc.* 39 599–605. 10.1249/mss.0b013e31802fa679 17414796

[B121] Shapira M. Licht A. Milman A. Pick C. G. Shohami E. Eldar-Finkelman H. (2007). Role of glycogen synthase kinase-3β in early depressive behavior induced by mild traumatic brain injury. *Mol. Cell. Neurosci.* 34 571–577. 10.1016/j.mcn.2006.12.006 17289399

[B122] Shively S. B. Perl D. P. (2012). Traumatic brain injury, shell shock, and posttraumatic stress disorder in the military—past, present, and future. *J. Head Trauma Rehabil.* 27 234–239. 10.1097/htr.0b013e318250e9dd 22573042

[B123] Shu I.-W. Onton J. A. O’Connell R. M. Simmons A. N. Matthews S. C. (2014). Combat veterans with comorbid PTSD and mild TBI exhibit a greater inhibitory processing ERP from the dorsal anterior cingulate cortex. *Psychiatry Res. Neuroimaging* 224 58–66. 10.1016/j.pscychresns.2014.07.010 25150386

[B124] Silverberg N. D. Panenka W. J. (2019). Antidepressants for depression after concussion and traumatic brain injury are still best practice. *BMC Psychiatry* 19:100. 10.1186/s12888-019-2076-9 PMC6438030 30917802

[B125] Slobounov S. M. Walter A. Breiter H. C. Zhu D. C. Bai X. Bream T. (2017). The effect of repetitive subconcussive collisions on brain integrity in collegiate football players over a single football season: a multi-modal neuroimaging study. *NeuroImage Clin.* 14 708–718. 10.1016/j.nicl.2017.03.006 28393012 PMC5377433

[B126] Staib J. M. Valle R. D. Knox D. K. (2018). Disruption of medial septum and diagonal bands of Broca cholinergic projections to the ventral hippocampus disrupt auditory fear memory. *Neurobiol. Learn. Mem.* 152 71–79. 10.1016/j.nlm.2018.05.009 29783059 PMC6000831

[B127] Stein D. G. (2013). A clinical/translational perspective: can a developmental hormone play a role in the treatment of traumatic brain injury? *Hormones Behav.* 63 291–300. 10.1016/j.yhbeh.2012.05.004 22626570

[B128] Sufrinko A. M. Mucha A. Covassin T. Marchetti G. Elbin R. J. Collins M. W. (2017). Sex differences in vestibular/ocular and neurocognitive outcomes after sport-related concussion. *Clin. J. Sport Med.* 27 133–138. 10.1097/jsm.0000000000000324 27379660 PMC5203982

[B129] Sussman E. S. Pendharkar A. V. Ho A. L. Ghajar J. (2018). Chapter 3 – Mild traumatic brain injury and concussion: terminology and classification. *Handb. Clin. Neurol.* 158 21–24. 10.1016/B978-0-444-63954-7.00003-3 30482349

[B130] Szczupak M. Hoffer M. Murphy S. Balaban C. (2016). “Posttraumatic dizziness and vertigo,” in *Handbook of Clinical Neurology Neuro-Otology*, eds Furman J. M. Lempert T. (Amsterdam: Elsevier). 10.1016/B978-0-444-63437-5.00021-2 27638079

[B131] Tapp Z. M. Godbout J. P. Kokiko-Cochran O. N. (2019). A tilted axis: maladaptive inflammation and HPA axis dysfunction contribute to consequences of TBI. *Front. Neurol.* 10:345. 10.3389/fneur.2019.00345 PMC6491704 31068886

[B132] Tate D. F. Wade B. S. C. Velez C. S. Drennon A. M. Bolzenius J. Gutman B. A. (2016). Volumetric and shape analyses of subcortical structures in United States service members with mild traumatic brain injury. *J. Neurol.* 263 2065–2079. 10.1007/s00415-016-8236-7 27435967 PMC5564450

[B133] Terry D. P. Mewborn C. M. Miller L. S. (2019). Repeated sport-related concussion shows only minimal white matter differences many years after playing high school football. *J. Int. Neuropsychol. Soc.* 25 950–960. 10.1017/s1355617719000754 31383046

[B134] Teutsch P. Jones C. E. Kaiser M. E. Gardner N. A. Lim M. M. (2018). Gait and conditioned fear impairments in a mouse model of comorbid TBI and PTSD. *Behav. Neurol.* 2018 1–10. 10.1155/2018/6037015 30327687 PMC6171258

[B135] Teymoori A. Gorbunova A. Haghish F. E. Real R. Zeldovich M. Wu Y.-J. (2020). Factorial structure and validity of depression (phq-9) and anxiety (gad-7) scales after traumatic brain injury. *J. Clin. Med.* 9:873. 10.3390/jcm9030873 32210017 PMC7141536

[B136] Uddin O. Studlack P. E. Parihar S. Keledjian K. Cruz A. Farooq T. (2019). Chronic pain after blast-induced traumatic brain injury in awake rats. *Neurobiol. Pain* 6:100030. 10.1016/j.ynpai.2019.100030 31223145 PMC6565615

[B137] Vargas G. Rabinowitz A. Meyer J. Arnett P. A. (2015). Predictors and prevalence of postconcussion depression symptoms in collegiate athletes. *J. Athletic Training* 50 250–255. 10.4085/1062-6050-50.3.02 25643158 PMC4477919

[B138] Waid-Ebbs J. K. Daly J. Wu S. S. Berg W. K. Bauer R. M. Perlstein W. M. (2014). Response to Goal Management Training in Veterans with blast-related mild traumatic brain injury. *J. Rehabil. Res. Dev.* 51 1555–1566. 10.1682/jrrd.2013.12.0266 25860148

[B139] Weber J. T. (2007). Experimental models of repetitive brain injuries. *Prog. Brain Res. Neurotrauma New Insights Pathol. Treatment* 161 253–261. 10.1016/s0079-6123(06)61018-2 17618983

[B140] Wojtowicz M. Gardner A. J. Stanwell P. Zafonte R. Dickerson B. C. (2018). Iverson GL: cortical thickness and subcortical brain volumes in professional rugby league players. *NeuroImage Clin.* 18 377–381. 10.1016/j.nicl.2018.01.005 29487794 PMC5814377

[B141] Wright D. K. Obrien T. J. Shultz S. R. Mychasiuk R. (2017). Sex matters: repetitive mild traumatic brain injury in adolescent rats. *Ann. Clin. Transl. Neurol.* 4 640–654. 10.1002/acn3.441 28904986 PMC5590540

[B142] Wunderle K. Hoeger K. M. Wasserman E. Bazarian J. J. (2014). Menstrual phase as predictor of outcome after mild traumatic brain injury in women. *J. Head Trauma Rehabil.* 29 E1–E8. 10.1097/HTR.0000000000000006 PMC5237582 24220566

[B143] Xiong Y. Mahmood A. Chopp M. (2013). Animal models of traumatic brain injury. *Nat. Rev. Neurosci.* 14 128–142. 23329160 10.1038/nrn3407 PMC3951995

